# Factors influencing treatment decisions in HIFU treatment of adenomyosis: A retrospective study

**DOI:** 10.3389/fsurg.2022.941368

**Published:** 2022-10-12

**Authors:** Qiao Zhong, Mei-Jie Yang, Yan Hu, Li Jiang, Jing-Wen Yu, Jin-Yun Chen, Wen-Zhi Chen

**Affiliations:** ^1^ State Key Laboratory of Ultrasound in Medicine and Engineering, College of Biomedical Engineering, Chongqing Medical University, Chongqing, China; ^2^ College of Medical Informatics, Chongqing Medical University, Chongqing, China; ^3^ Department of Oncology, First Affiliated Hospital of Chongqing Medical University, Chongqing, China

**Keywords:** adenomyosis, HIFU, dysmenorrhea, menorrhagia, decision-making

## Abstract

**Objective:**

To explore the influencing factors of decision-making in patients with adenomyosis, who are receiving high-intensity focused ultrasound (HIFU) treatment.

**Methods:**

A total of 776 patients with adenomyosis were enrolled into HIFU group (241 cases) and hysterectomy group (535 cases) according to the treatment methods. The general data, clinical symptoms, marital and childbearing history, and economic status were compared between the two groups, and factors with *P *< 0.05 were introduced into multivariate logistic regression analysis to determine the determinants of patients choosing HIFU.

**Results:**

The average age of the patients in the HIFU group was 39.1 ± 5.2 years, which was lower than that in the hysterectomy group, which was 45.1 ± 3.9 years (*P *< 0.05). The basic medical insurance for urban workers in the HIFU group was more than the hysterectomy group (*P *< 0.05). 95.9% of the hysterectomy group had no desire to have children, compared to 60.6% of the HIFU group, the difference was significant (*P *< 0.05). The treatment costs of HIFU group were significantly lower than that of hysterectomy group (*P *< 0.05). The main symptoms of the two groups were dysmenorrhea, menorrhagia, and secondary anemia. The results of multivariate logistic regression analysis showed that 31–40 years old, fertility desire, dysmenorrhea, menorrhagia, anemia and dizziness and fatigue were the influencing factors for the decision-making of HIFU for patients with adenomyosis.

**Conclusion:**

31–40 years old, fertility desire, dysmenorrhea, menorrhagia, anemia and dizziness and fatigue were the influencing factors for patients to choose HIFU treatment. HIFU therapy has emerged as a new option for patients with adenomyosis as an alternative to hysterectomy.

## Introduction

Adenomyosis (AM) is a common benign gynecological disease, defined as the presence of endometrial glands and stroma deep within the myometrium, causing myometrial hypertrophy and hyperplasia (1, 2). The true prevalence of adenomyosis is difficult to determine; for decades, the diagnosis of adenomyosis has been made mainly by pathology after hysterectomy, with a prevalence of about 5% to 70% (3). Over the past decade, adenomyosis can also be diagnosed without surgical intervention. Advances in imaging technology have created the possibility of non-invasive diagnosis of adenomyosis, such as magnetic resonance imaging (MRI) and transvaginal ultrasonography (TVUS). This allows the diagnosis to be made in young women, with or without clinical symptoms (4–7). Clinically, adenomyosis can result in abnormal bleeding, pelvic pain, and infertility, although approximately 30% of patients are asymptomatic (8–10). There are two types of adenomyosis: diffuse and focal (when a definite nodule is found, it can be called an adenomyoma). In addition, adenomyosis is often combined with other gynecological diseases, such as endometriosis (11, 12) and leiomyoma (13).

Adenomyosis treatment includes drug therapy, conservative surgery, interventional therapy, and hysterectomy. Hysterectomy is currently considered to be the last resort for adenomyosis (14)^,^ but it is a difficult choice for patients who want to preserve their uterus or maintain fertility. Unfortunately, approximately 50% of patients will experience relapse after conservative surgery (15). Drug therapy can effectively relieve clinical symptoms such as dysmenorrhea and menorrhagia (16).

High-intensity focused ultrasound (HIFU) is a new technology developed in recent years for non-invasive local hyperthermia treatment of tumors. It is widely used in gynecological diseases and has a good curative effect on uterine fibroids and adenomyosis (17, 18). The therapeutic mechanism of HIFU therapy is to focus the ultrasound beam generated by an external transducer on the target lesion in the body. The mechanical effect of ultrasound is transformed into thermal effect and cavitation effect. The purpose of HIFU in the treatment of adenomyosis is to selectively ablate adenomyosis lesions to relieve the symptoms. It relies on highly focused ultrasound energy to precisely destroy uterine adenomyosis in a non-invasive, bloodless manner (19). Patients treated with HIFU had significantly fewer symptoms of adenomyosis and recovered at a relatively high pregnancy rate after the treatment (20, 21). However, due to the unclear boundary of adenomyosis, some patients' symptom relief only lasts for a certain period, Liu et al. showed that 26% of patients relapsed after HIFU treatment, and the median time to relapse was 12 months (22). It should be noted that HIFU treatment is mainly suitable for symptomatic patients with lesions larger than 3 cm (23, 24).

Currently, there is no guideline for the management of adenomyosis, especially the uterine-sparing treatment of these adenomyosis presents formidable challenges to the gynecologist. Therefore, this study aimed to explore the factors influencing a patient's decision to undergo HIFU by extracting raw medical records, which can provide a basis for exploring optimized treatment strategies and clinical management options for patients with adenomyosis.

## Materials and methods

### Study design

**Figure 1:**
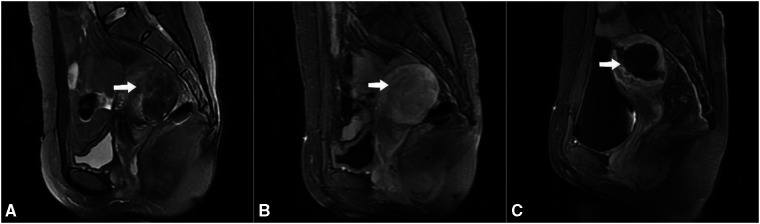
Mr images obtained from a patient with adenomyosis. (A) T2WI shows a well-defined adenomyosis lesion, which is a poorly defined low-signal lesion with scattered punctate and patchy high-signal foci located in the posterior uterine wall (arrow); (B) Contrast-enhanced MRI showed perfusion of the adenomyotic lesion (arrow); (C) Post-treatment T1-weighted contrast-enhanced image shows that the non-perfused volume ratio was 83.5% (arrow).

This cross-sectional retrospective study was conducted at the First Hospital of Chongqing Medical University. We reviewed all patients with adenomyosis (ICD-10: n80.001) who were admitted to hospital and received treatment from January 2013 to December 2020. Inclusion criteria were as follows: (1) premenopausal women; (2) typical symptoms: dysmenorrhea, menorrhagia; (3) diagnosis of adenomyosis by clinical evaluation, vaginal ultrasound and/or MRI (Figure 1).

The exclusion criteria were: (1) no intervention on the day of admission and discharge; (2) incomplete electronic medical record description information; (3) postmenopausal women with abnormal uterine bleeding or/and lower abdominal pain; (4) patients who received treatments other than hysterectomy and HIFU; (5) patients with suspected or confirmed uterine malignancy; (6) patients with acute pelvic inflammatory disease.

After deliberation by the ethics committee of Chongqing Medical University (Ethics Approval Number, 2021–006), it is unnecessary to sign the informed consent form. However, it shall be implemented in strict accordance with the general provisions of the Declaration of Helsinki. Besides, all patient information is strictly confidential.

### Data extraction

The age of each patient was calculated from the date of birth recorded in the case record to the date of hospitalization. At the same time, the clinical data of all patients were collected through a medical history system. These data included the patients' age, reproductive desire, work status, symptoms, marital status, abortion, deliver, cesarean section, medical insurance, and medical expenses.

### Statistical analysis

SPSS 22.0 statistical software (IBM SPSS, USA) was used for the data analysis. Quantitative data are presented as mean ± SD, while count data are presented as [n (%)]. The *χ*2 test was used to compare differences between groups and *P *< 0.05 was considered statistically significant. In addition, multivariate logistic regression analysis was used to determine the influencing factors of patients' choice of HIFU.

## Results

There was a total of 973 patients diagnosed with adenomyosis on admission, excluding 127 patients who received drug treatment, 42 patients who received lesion resection, and 29 patients with abnormal uterine bleeding or lower abdominal pain in postmenopausal women. The remaining 776 patients, including 535 (57.5%) patients who underwent hysterectomy and 241 (42.5%) who underwent HIFU treatment, were included in this study.

### Adenomyosis-related symptoms

**Figure 2:**
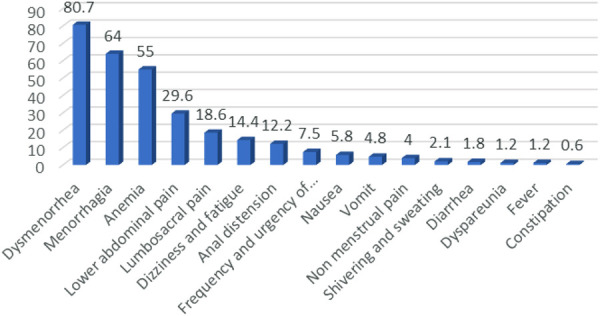
Symptoms of patients with adenomyosis (%).

Among the 776 patients, 626 (80.7%) complained of dysmenorrhea, 497 (64%) menorrhagia, 427 (55%) secondary anemia and 230 (29.6%) lower abdominal pain (Figure 2).

### General conditions of patients and treatment decisions

The patients were enrolled into two groups according to the treatment method. The mean age of patients in the hysterectomy group was 45.1 ± 3.9 years (range = 31–52 years), and the HIFU group was 39.1 ± 5.2 years (range = 25–52 years), the difference was significant (t = 17.704 *P* < 0.001).60.6% of the HIFU group were younger than 40 years old, and only 11.6% were in the hysterectomy group. There was a statistically significant difference in age distribution between the two groups (*χ*2 = 211.722, *P *< 0.001). Meanwhile, the HIFU group had a desire to have children in 60.6%, which was significantly higher than the hysterectomy group (4.1%) (*χ*2 = 312.346, *P *< 0.001).In addition, patients with jobs in the HIFU group accounted for 74.4%, which was significantly higher than that in the hysterectomy group (50.3%); while 23.7% of the patients in the HIFU group had temporary job, which was significantly lower than that in the hysterectomy group (41.1%) (*χ*2 = 42.656, *P *< 0.001) (Table 1).

**Table 1:** Comparison of basic conditions of patients with adenomyosis [*n*(%)].

	HIFU (*n* = 241)	Hysterectomy (*n* = 535)	*P* value
Age			0.000
≤30 years	12 (5)	0 (0)	
31–40 years	134 (55.6)	62 (11.6)	
41–50 years	93 (38.6)	432 (80.7)	
> 50 years	2 (0.8)	41 (7.7)	
Reproductive desire			0.000
Yes	146 (60.6)	22 (4.1)	
No	95 (39.4)	513 (95.9)	
Work status			0.000
Patients with jobs	180 (74.7)	275 (51.4)	
Temporary job	58 (23.7)	220 (41.1)	

### Adenomyosis-related symptoms and treatment decisions

The main clinical manifestations of hospitalized patients were dysmenorrhea, menorrhagia, secondary anemia, and lower abdominal pain. Compared with the two treatment decisions, dysmenorrhea accounted for 90.1% in the HIFU group, which was significantly higher than that in the hysterectomy group (75.9%) (*χ*2 = 21.470, *P *< 0.001); Menorrhagia in the HIFU group were 52.1%, which was significantly lower than that in the hysterectomy group (69.3%) (*χ*2 = 21.009, *P *< 0.001);The secondary anemia covered 38.8% in the HIFU group, which was significantly lower than that in the hysterectomy group (61.9%) (*χ*2 = 38.161, *P *< 0.001).At the same time, other symptoms included dizziness and fatigue, urinary frequency and urgency, nausea, vomiting, diarrhea and dyspareunia, and the difference between the two groups was statistically significant (all *P *< 0.05) (Table 2).

**Table 2:** Comparison of related symptoms in patients with adenomyosis [*n*(%)].

	HIFU	Hysterectomy	*P* value
Dysmenorrhea	218 (90.5)	408 (76.3)	0.000
Menorrhagia	126 (52.3)	371 (69.3)	0.000
Anemia	93 (38.6)	334 (62.4)	0.000
Lower abdominal pain	63 (26.1)	167 (31.2)	0.174
Lumbosacral pain	46 (19.1)	98 (18.3)	0.842
Dizziness and fatigue	14 (5.8)	98 (18.3)	0.000
Anal distension	33 (13.7)	62 (11.6)	0.409
Frequency and urgency of micturition	13 (5.4)	45 (8.4)	0.184
Nausea	22 (9.1)	23 (4.3)	0.012
Vomit	21 (8.7)	16 (3)	0.001
Non menstrual pain	8 (3.3)	23 (4.3)	0.692
Shivering and sweating	6 (2.5)	10 (1.9)	0.590
Diarrhea	9 (3.7)	5 (0.9)	0.015
Dyspareunia	5 (2.1)	4 (0.7)	0.145
Fever	1 (0.4)	8 (1.5)	0.287
Constipation	1 (0.4)	4 (0.7)	1.000

### Marital and reproductive Status and treatment decisions

The patients who have childbirth history of the HIFU group was 79.3%, which was lower than that of the hysterectomy group (92.3%), and the difference was statistically significant (*χ*2 = 27.475, *P *< 0.001). There was no significant difference in marital status, history of abortion, and childbirth between the two groups (*P *> 0.05) (Table 3).

**Table 3:** Comparison of marital and reproductive status of patients with adenomyosis. [*n*(%)].

	HIFU	Hysterectomy	*P* value
Marital status			0.384
Married	230 (95.4)	518 (96.8)	
Divorce	9 (3.7)	16 (3)	
Unmarried	2 (0.8)	1 (0.2)	
Abortion	209 (89)	476 (89)	0.367
Deliver	191 (79.3)	494 (92.3)	0.000
Cesarean section	56 (23.2)	115 (21.5)	0.588

### Economic situation and treatment decisions

The basic medical insurance for urban workers in the HIFU group (71.4%) was higher than that in the hysterectomy group (53.6%), while the basic medical insurance for urban residents in the HIFU group (24.5%) was lower than that in the hysterectomy group (40.7%). (*χ*2 = 25.793, *P *< 0.001). The proportion of HIFU treatment costs less than ¥25,000 was 62.2%, while that in the hysterectomy group was 19.4%. There was a statistically significant difference in the distribution of treatment costs between the two groups, and the hysterectomy group was higher (*χ*2 = 230.024, *P *< 0.001) (Table 4).

**Table 4:** Comparison of economic situation of patients with adenomyosis [*n*(%)].

	HIFU	Hysterectomy	*P* value
Medical insurance			0.000
Basic medical insurance for employee	172 (71.4)	287 (53.6)	
Basic medical insurance for residents	60 (24.9)	233 (43.5)	
Commercial medical insurance	9 (3.7)	15 (2.8)	
Treatment costs (CNY)			0.000
≤15,000	80 (33.2)	7 (1.3)	
15,000–25,000	70 (29)	86 (18.1)	
25,000–50,000	91 (37.8)	342 (63.9)	
> 50,000	0 (0)	100 (18.7)	

### Factors influencing HIFU treatment decision-making

Univariate analysis was performed on the influence of patients' general information, clinical symptoms, marriage and childbearing history, economic status, etc. on treatment decisions, and statistically significant indicators were included in multivariate analysis, including age, medical insurance category, work status, reproductive requirements, and birth history., dysmenorrhea, menorrhagia, anemia, nausea, vomiting, diarrhea, dizziness and fatigue, *P* < 0.05 was used to screen the patients with adenomyosis Influencing factors for choosing HIFU.

The results showed that between 31 and 40 years old (OR = 0.188, *P *= 0.039), fertility requirements (OR = 0.051, *P *< 0.001), dysmenorrhea (OR = 0.525, *P *= 0.035), menorrhagia (OR = 1.605, *P *= 0.048), secondary anemia (OR = 1.799, *P *= 0.013), and dizziness and fatigue (OR = 2.453, *P *= 0.023) were the influencing factors for HIFU decision-making in patients with adenomyosis (Table 5).

**Table 5:** Factors influencing decision-making for HIFU treatment [*n*(%)].

	OR	OR (95% CI)	*P* value
31–40 years old	0.188	0.038-0.920	0.039
Reproductive desire	0.051	0.025-0.104	0.000
Dysmenorrhea	0.525	0.288-0.956	0.035
Menorrhagia	1.605	1.005-2.565	0.048
Anemia	1.799	1.129-2.866	0.013
Dizziness and fatigue	2.453	1.134-5.306	0.023

## Discussion

Adenomyosis is a common chronic gynecological disease. Treating adenomyosis remains a global challenge, and most women want to protect their uterus while effectively relieving symptoms and improving their quality of life. The problem that this study attempts to solve is to find out the characteristics of the population who choose HIFU treatment and hysterectomy treatment, and the factors influencing the decision-making of patients choosing HIFU treatment. The results show that patients under the age of 40, who have jobs, have basic medical insurance for urban workers, and have fertility requirements are more inclined to choose HIFU; However, patients who are older than 40 years old, have no fixed jobs, have basic medical insurance for urban residents, and have no desire to have children tend to choose hysterectomy. In addition, the HIFU group had fewer treatment costs compared to hysterectomy.

In clinical practice, we found that many factors may affect patients' choice for HIFU treatment. In this study, patients treated with HIFU were significantly younger than the hysterectomy group (*P *< 0.001), with a range of age 31–40 years (55.6%), meanwhile there was also a statistical difference in fertility requirements between patients treated with HIFU and the hysterectomy group (*P* < 0.001), with 60.6% of patients treated with HIFU having fertility requirements. More and more young women were diagnosed, mainly because the diagnosis now was based on imaging diagnoses such as TVUS and MRI (9, 20). At the same time, with the change in women's concept of fertility, more and more women choose to give birth after the age of 30 and retain the uterus, and symptom relief are their primary concerns when seeking treatment options. Therefore, as a non-invasive treatment, HIFU has become the preferred treatment method for women of childbearing age between 31 and 40 years old. There are multiple clinical case reports of improved fertility after HIFU treatment (25, 26). The benefit of HIFU treatment for patients with adenomyosis deserves further study.

Severe dysmenorrhea and menorrhagia are considered as the most important symptoms affecting patients' quality of life. The main purpose of adenomyosis treatment is to relieve symptoms and improve the quality of life of patients. HIFU has been used to treat adenomyosis for many years. Recently, more and more gynecologists in China consider HIFU as a routine treatment for patients with adenomyosis. The results of 3–12 months' follow-up of patients with adenomyosis treated with HIFU showed that the clinical effective rate of improving dysmenorrhea or menorrhea was about 80% (18, 27). And combined treatment regimen with GnRHa and LNG-IUS after HIFU can significantly improve long-term outcomes (28). Although hysterectomy can also solve the patient's symptom problems, it brings many serious side effects, such as loss of reproductive function, impaired pelvic anatomical integrity, impaired neural network systems, and the gonadal endocrine axis (29, 30). Therefore, hysterectomy is mainly suitable for older patients, who have no fertility requirements, severe symptoms, and poor drug treatment effects or drug contraindications. HIFU may be a better choice for patients who have fertility requirements and want to relieve symptoms. However, HIFU technology as a way to treat adenomyosis, its comprehensive management plan which needs further study, to improve its long-term efficacy and reduce recurrence.

Another important finding of this study was that work status and medical insurance category were significantly associated with the choice of HIFU treatment in patients. 74.7% of the patients who received HIFU treatment were working and had a high level of basic medical insurance for urban workers. The main reason may be that the Patients with jobs have less time controllability than who with temporary job. Considering the postoperative recovery time, HIFU has more advantages than hysterectomy. Another aspect is that the proportion of anyone at work who have received higher education is relatively high, they have a deeper understanding of health awareness and medical knowledge and are more inclined to new technologies with fewer side effects (23). In addition, adenomyosis is still at risk of recurrence after HIFU treatment and the possibility of getting secondary interventions is high. Therefore, financial freedom and independent decision-making ability have become important factors affecting treatment decisions. Patients with jobs are more inclined to choose less traumatic and repeatable treatments.

The main concern of patients when they visit the clinic, besides the cure rate of the disease, is the actual cost of medical care. This study found that the medical cost of HIFU treatment was significantly lower than that of hysterectomy (*P* < 0.001), and 62.2% of patients spent less than 25,000 CNY. Therefore, HIFU is inexpensive than hysterectomy. Evaluating the cost of care not only helps healthcare providers and healthcare systems utilize limited healthcare resources, it also provides a way to maximize benefits to patients and healthcare systems.

This study is a retrospective study, there is no unified path design for patient selection, and it is difficult to avoid the influence of physicians' own technical knowledge on patients' treatment choices. Due to the possibility of skin and subcutaneous tissue burns during HIFU treatment, the limitation of large abdominal scarring in the path of the ultrasound beam remains (31). Although research has shown that scar patches are safe and effective in relieving abdominal scarring in patients with uterine and adenomyosis treated with MRgHIFU (32). But this study did not consider the effect of this factor on treatment decisions in patients with adenomyosis, which is one of the study's limitations.

## Conclusion

The results of this study show that HIFU is safe and effective in the treatment of adenomyosis, and the cost is lower than that of hysterectomy. Age 31–40, fertility desire, dysmenorrhea, menorrhagia, anemia, and dizziness and fatigue were the factors influencing patients' treatment decision to choose HIFU. In conclusion, HIFU treatment can keep the uterus while relieving symptoms. It has become one of the new choices for patients with adenomyosis, and younger (31–40 years old) working patients who are willing to choose HIFU treatment. Meanwhile, prospective studies on patient self-report scales are needed in the future to optimize patient treatment decisions.

## Data Availability

The raw data supporting the conclusions of this article will be made available by the authors, without undue reservation.
